# Beyond Glycemic Control: GLP-1RA–Based Therapies and Emerging Targets Beyond the Metabolic Axis

**DOI:** 10.3390/jcm15072786

**Published:** 2026-04-07

**Authors:** Wojciech Matuszewski, Katarzyna Wołos-Kłosowicz, Paulina Włodarczyk, Patrycja Waśniewska, Robert Modzelewski, Jan Marek Górny, Michał Szklarz, Mikołaj Madeksza, Judyta Juranek

**Affiliations:** 1 Clinic of Endocrinology, Diabetology and Internal Medicine, School of Medicine, Collegium Medicum, University of Warmia and Mazury, 10-719 Olsztyn, Poland; kat.wolos@gmail.com (K.W.-K.); mikolaj.madeksza@icloud.com (M.M.); 2 Department of Human Physiology and Pathophysiology, University of Warmia and Mazury, 10-719 Olsztyn, Poland

**Keywords:** GLP-1 receptor agonists, mental health, addiction, cancer, polycystic ovary syndrome (PCOS), neuroprotection

## Abstract

**Background/Objectives**: Modern diabetes therapy extends beyond glycemic control and increasingly focuses on comprehensive risk reduction to prevent long-term complications, improve quality of life, and reduce premature mortality. Accordingly, modern therapeutic approaches address not only glucose metabolism but also cardiovascular, renal, and metabolic consequences of diabetes. Within this context, glucagon-like peptide-1 receptor agonists (GLP-1 RAs) have emerged as a significant therapeutic class. In addition to their well-known effects on glycemic control and the metabolic-cardiovascular-renal axis, increasing evidence suggests that these agents may exert a range of pleiotropic effects and opening new therapeutic venues, discussed in this review. **Methods**: A narrative review of the literature was conducted using the PubMed, Scopus, and Google Scholar databases. Publications from 2014 and 2026 were screened using predefined keywords related to GLP-1 RAs and their potential effects across multiple physiological systems and diseases. Notably, more than 80% of the included studies were published between 2020 and 2026, reflecting the recent growth of research in this field. **Results**: GLP-1 RAs have been associated with beneficial effects across a wide range of conditions, including substance use disorders, mental health disorders, neurodegenerative diseases, obesity-related complications, liver disease, genitourinary disorders, osteoarthritis, and sleep apnea. While they are currently the most effective pharmacological agents for the treatment of obesity, they also significantly reduce hepatic steatosis and are associated with a decreased risk of developing hepatocellular carcinoma. Furthermore, they have also demonstrated positive effects against prostate cancer, polycystic ovary syndrome (PCOS), improved libido and fertility. **Conclusions**: GLP-1 RAs should no longer be regarded solely as antihyperglycemic agents. Instead, they represent a versatile therapeutic class with expanding clinical relevance across multiple medical disciplines. While current evidence is promising, further large-scale, well-designed clinical trials are required to define their full therapeutic potential.

## 1. Introduction

GLP-1 receptor agonists (GLP-1 RAs) constitute a class of medications originally developed for the treatment of type 2 diabetes mellitus (T2DM). The first drug in this class, exenatide, was approved by the U.S. Food and Drug Administration (FDA) in April 2005. Exenatide was derived from a naturally occurring peptide found in the saliva of the Gila monster (*Heloderma suspectum*), which exhibits properties similar to endogenous human GLP-1 [1,2] and requires twice-daily subcutaneous administration. Subsequently, longer-acting and more effective formulations were developed, including liraglutide (approved in 2009), followed by once-weekly agents such as dulaglutide (2014), semaglutide (2017), and tirzepatide (2023). Tirzepatide is a first-in-class, dual agonist that acts on both glucose-dependent insulinotropic polypeptide (GIP) receptors and glucagon-like peptide-1 (GLP-1) receptors [3]. Most GLP-1 RAs are administered subcutaneously, with the notable exception of semaglutide, which is also available in an oral formulation. Currently, GLP-1 RAs are approved for the treatment of T2DM and obesity. However, during their clinical use, numerous non-metabolic effects have been observed, including delayed progression of chronic kidney disease (CKD), reduction in proteinuria [4], lowered blood pressure, improved lipid profiles, and decreased risk of major adverse cardiovascular events (MACE) [5]. These pleiotropic properties have sparked considerable interest in their potential therapeutic applications beyond diabetes and obesity. One notable investigation in this area is the SELECT trial, a large international clinical study evaluating the effect of semaglutide on MACE incidence in individuals with overweight or obesity and established cardiovascular disease, but without diabetes. It enrolled 17,604 patients with a median follow-up of approximately 40 months. The trial demonstrated that semaglutide improved all outcome measures in patients with heart failure at random assignment compared with those without heart failure (hazard ratio (HR) 0.72, 95% CI 0.60–0.87 for MACE; 0.79, 0.64–0.98 for the heart failure composite endpoint; 0.76, 0.59–0.97 for cardiovascular death; and 0.81, 0.66–1.00 for all-cause death; all interaction *p* > 0.19) [6]. Importantly, the reduction in cardiovascular risk was evident shortly after treatment initiation, suggesting that semaglutide’s benefits may extend beyond weight loss alone. The favorable cardiovascular effects of incretin-based therapies, such as tirzepatide, were also confirmed in the randomized SUMMIT trial, which evaluated tirzepatide in 731 patients with obesity and heart failure with preserved ejection fraction (HFpEF). Participants were randomly assigned to receive tirzepatide or placebo at 129 centers in nine countries between 20 April 2021 and 30 June 2023, with follow-up continuing until the final study visit on 2 July 2024. Treatment with tirzepatide was associated with a 38% reduction in the risk of cardiovascular death or worsening heart failure and a 56% reduction in hospitalizations for heart failure compared with placebo [3,5,7]. GLP-1 RAs have also been shown to exert broader pharmacological effects, including anti-inflammatory, neuroprotective, antihypertensive, and lipotoxicity-reducing properties. These effects are relevant to both physiological and pathological processes implicated in AD, hypertension, and metabolic dysfunction-associated steatohepatitis (MASH). Although the underlying mechanisms are not fully understood, further studies may facilitate the identification of novel therapeutic targets for AD, hypertension, and metabolic dysfunction–associated steatohepatitis [8].

The aim of this paper is to present the current state of knowledge regarding the pleiotropic effects of GLP-1 RAs and to explore their potential applications beyond glycemic control and weight management. Expanding the therapeutic use of these agents may represent a significant advancement in the treatment and prevention of various diseases, particularly among patients with metabolic syndrome.

## 2. Materials and Methods

A narrative literature review was conducted using PubMed, Scopus, and Google Scholar to identify studies evaluating the pleiotropic effects of GLP-1 RAs. Publications published between 2014 and March 2026 were included in the search.

Original research articles, randomized clinical trials, cohort studies, and relevant mechanistic studies were considered. Narrative reviews and meta-analyses were also screened to identify additional relevant references. Publications not available in English, case reports, and articles not directly related to GLP-1 RAs were excluded from the analysis. Studies were prioritized based on relevance to the discussed outcomes and methodological quality. 

The review aimed to highlight representative and influential evidence across different clinical domains rather than to systematically capture all available studies. The literature search strategy and eligibility criteria are summarized in Table 1.

## 3. Results and Discussion

### 3.1. Mechanism of Action and Physiological Effects of GLP-1 Ras

GLP-1 is an incretin hormone secreted by enteroendocrine L cells of the small intestine in response to nutrient intake. After release, GLP-1 binds to the GLP-1 receptor (GLP-1R), a G-protein-coupled receptor expressed in pancreatic β-cells and in several extra-pancreatic tissues. Activation of GLP-1R stimulates adenylate cyclase and increases intracellular cAMP, thereby enhancing glucose-dependent insulin secretion. Beyond its pancreatic effects, GLP-1 slows gastric emptying and acts on hypothalamic pathways regulating appetite and satiety, leading to reduced food intake and weight loss.

However, endogenous GLP-1 has a very short biological half-life due to rapid degradation by the enzyme dipeptidyl peptidase-4 (DPP-4). GLP-1 RAs were therefore developed to reproduce the physiological actions of GLP-1 while resisting DPP-4-mediated degradation, allowing sustained receptor activation and enhanced therapeutic efficacy.

### 3.2. Established Mechanisms and Clinical Effects of GLP-1 RAs

GLP-1 RAs currently represent one of the most extensively evaluated pharmacological classes in the management of metabolic disorders. Their core mechanisms of action have been rigorously validated across both experimental and clinical trials, providing a robust physiological foundation for their therapeutic application. Specifically, their efficacy in modulating glycemic homeostasis, body weight, gastrointestinal motility, and cardiovascular function is well-established.

Table 2 shows the primary, evidence-based pharmacological effects of GLP-1 RAs as documented in the current scientific literature and confirmed by landmark clinical trials. These established mechanisms serve as a benchmark for further exploration of the potential pleiotropic effects associated with this drug class.

### 3.3. Pleiotropic Effects of GLP-1 Receptor Agonists

#### 3.3.1. GLP-1 RAs on Alcohol Use

According to the 2022 data from the National Survey on Drug Use and Health, alcohol remains the most commonly used psychoactive substance in the United States. Alcohol Use Disorder (AUD) is a chronic condition associated with significant medical, social, and psychological consequences [27]. Therefore, the search for new therapeutic strategies remains ongoing. GLP-1 RAs have attracted increasing attention from the scientific community, particularly due to their effects on the central nervous system and their potential application in addiction treatment.

GLP-1 RAs play an important role in modulating the activity of the brain’s reward system, which may represent a novel therapeutic target for substance use disorders [28]. The reward system comprises a network of neural structures responsible for motivation and behavior regulation, activated in response to satisfying needs and receiving pleasurable stimuli. Psychoactive substances such as alcohol, opioids, and amphetamines strongly stimulate this system, contributing to the development of addiction [28,29].

Tirzepatide, a dual agonist of the GLP-1 and GIP receptors, influences central nervous system pathways involved in mood regulation, reward processing, and addictive behavior. The GLP-1 receptor plays a key role in the functioning of the reward system, and its activation by tirzepatide may modulate dopaminergic signaling, which is critical in the rewarding effects of food and alcohol [30].

Preclinical studies conducted by Jerlhag E et al. demonstrated that GLP-1 RAs reduce alcohol intake in rats. This effect is achieved, in part, through inhibition of dopamine release in the nucleus accumbens—a structure central to the brain’s reward circuitry. Experiments with semaglutide confirmed these findings, showing both reduced alcohol consumption and prevention of relapse [31,32].

A randomized clinical trial led by Christian S. Hendershot evaluated the effect of once-weekly subcutaneous semaglutide administration on alcohol intake in individuals with AUD. The study enrolled 48 participants—34 women and 14 men—with a mean age of 39.9 years. Most participants had a body mass index (BMI) exceeding 30. The results demonstrated that semaglutide treatment led to a reduction in alcohol use, both in terms of total alcohol consumption and peak breath alcohol concentration [27]. Despite promising results, the small number of participants (*n* = 48) restricts the findings to a hypothesis-generating level.

The 12-month follow-up period retrospective cohort study, including electronic health records of 83,825 patients with obesity, demonstrated that semaglutide (*n* = 45,797) compared with other anti-obesity medications (*n* = 38,028) is associated with a 50–56% lower risk for both the incidence and recurrence of alcohol use disorder (0.37% vs. 0.73%; HR: 0.50, 95% CI: 0.39–0.63), consistent across gender, age group and race. Similar findings are replicated in the study population with 598,803 patients with T2DM [33].

Despite these promising findings from both preclinical and early clinical studies, the efficacy of GLP-1 RAs in treating AUD requires further investigation. While current observational and pilot data suggest a strong association, they are primarily hypothesis-generating; they do not confirm causality and require cautious interpretation. Large-scale, randomized, multicenter clinical trials involving more diverse patient populations are necessary to validate these results. Future research should prioritize the analysis of subgroup analyses based on gender, BMI, and other potential confounding variables [34]. While basic research must continue to explore underlying mechanisms, future clinical trials should focus on the safety, efficacy, and optimal dosing of GLP-1RAs, including their potential in combination with established pharmacological and behavioral therapies [35].

#### 3.3.2. GLP-1 RAs on Nicotine Use

Cigarette smoking remains the leading preventable cause of premature death. Despite numerous support programs, smoking cessation rates remain low. One of the key barriers is the occurrence of nicotine withdrawal symptoms and the associated weight gain following smoking cessation. This unfavorable metabolic consequence represents a significant deterrent for many smokers attempting to quit or maintain abstinence. Weight gain is particularly prominent during the first months of cessation, with an average increase of approximately 2.3 kg within the first two months [36].

GLP-1 RAs reduce caloric intake and body weight and have demonstrated potential in modulating addictive behaviors through their impact on the central reward system. They regulate synaptic dopamine availability and expression of the dopamine transporter (DAT), which plays a crucial role in motivation and reward-driven behaviors. Their influence on nicotine dependence also appears to involve the interpeduncular nucleus, a brain region implicated in nicotine withdrawal and craving [37].

Preclinical studies have shown that GLP-1 RAs reduce nicotine self-administration and nicotine-seeking behavior in animal models. Notably, in some cases, these effects persisted even after discontinuation of substance exposure [36].

In an observational study involving 222,942 patients with T2DM treated with various antidiabetic medications (including insulin, metformin, DPP-4 inhibitors, SGLT-2 inhibitors, sulfonylureas, thiazolidinediones, and GLP-1 RAs—among them 5967 semaglutide users), semaglutide use was associated with a 32% lower risk of tobacco use disorder compared to insulin (HR 0.68 [95% CI, 0.63 to 0.74]). Patients receiving semaglutide also showed reduced need for pharmacological smoking cessation aids and less frequent use of smoking cessation counseling [38].

GLP-1 RAs may represent a novel therapeutic avenue to complement standard nicotine replacement therapies in individuals with nicotine dependence. These agents may not only assist in addressing the addiction itself but also mitigate cessation-related complications such as nicotine craving, withdrawal symptoms, weight gain, hyperphagia, and other metabolic disturbances.

#### 3.3.3. GLP-1 RAs on Substance Use Disorders—Cannabinoids and Cocaine

Substance use disorders (SUDs) represent an escalating public health challenge both nationally and globally. In the United States, there is a steady increase in the prevalence of drug dependence, as reflected in epidemiological data, as well as a rise in drug-related hospitalizations and overdose deaths [39,40]. In recent years, an increased share of juvenile offenses has been attributed to drug-related criminal activity [41,42].

According to the 2019 European School Survey Project on Alcohol and Other Drugs (ESPAD), Polish adolescents ranked among the top four in Europe in the use of new psychoactive substances (NPS, commonly referred to as “legal highs”) and were the leading group in amphetamine use [43]. Drug addiction is a debilitating and chronic condition characterized by a recurrent cycle of intoxication, craving, withdrawal, and relapse. Regardless of the substance involved, addiction leads to the development of neuroanatomical abnormalities accompanied by functional brain changes and behavioral disturbances [44].

Cocaine exerts its psychoactive effects primarily through activation of the mesolimbic dopaminergic pathway. Dopaminergic projections from the ventral tegmental area (VTA) to forebrain structures, including the nucleus accumbens (NAc), play a central role in cocaine reinforcement and the development of dependence [40]. Cocaine increases dopamine levels and prevents its reuptake in the brain, which over time disrupts neural receptor function [45].

GLP-1 RAs may reduce cocaine dependence by acting on several brain regions involved in addiction processes, such as the lateral septum, dorsolateral and ventral tegmental areas, nucleus of the solitary tract, and nucleus accumbens [40].

In a mouse study conducted by Emil Egecioglu et al., systemic administration of exenatide inhibited cocaine uptake and expression in the central nervous system, leading to a reduction in cocaine-induced locomotor stimulation. A similar effect was observed in response to amphetamine administration [46].

Further preclinical studies by Chaves Filho et al. demonstrated potential antimanic or mood-stabilizing effects of liraglutide in D-amphetamine-induced mania models in mice. Liraglutide mitigated amphetamine-related cognitive deficits in certain behavioral tasks, effectively counteracting hyperlocomotion and impairments in spatial and executive memory [47].

The study involving approximately 700,000 individuals (85,223 patients with obesity who were prescribed semaglutide or non-GLP-1RA anti-obesity medications, with the findings replicated in 596,045 patients with T2DM) revealed that patients who initiated semaglutide therapy were found to have a 44% lower risk of incident cannabis use disorder (CUD) in patients with no prior history (HR: 0.56, 95% CI: 0.42–0.75) and a 38% lower risk of recurrent CUD in those with a history of the disorder (HR: 0.62, 95% CI: 0.46–0.84). These findings were consistent across age, gender, and race, suggesting that GLP-1 RAs may play a transformative role in modulating brain reward pathways and treating diverse addictions. However, further preclinical studies are warranted to understand the underlying mechanism and randomized clinical trials are needed to support its use clinically for CUD [48].

There is also growing speculation that GLP-1 RAs may reduce various forms of compulsive consumption—such as compulsive shopping or gambling—through their influence on the reward system. However, further research is required to clarify these potential effects [49].

#### 3.3.4. GLP-1 RAs on Opioid Use

The misuse of opioid analgesics has become a pressing contemporary social and medical issue. This problem is fueled by inappropriate use of opioids—either outside the framework of the WHO analgesic ladder or for recreational purposes—due to their euphoric properties. With the aging population steadily increasing, there is a growing susceptibility to chronic pain syndromes, often associated with multiple comorbidities. Chronic pain not only reduces quality of life but also poses a therapeutic challenge for healthcare providers [50].

In many cases, opioid therapy remains the only viable option for improving patients’ daily functioning and overall well-being. However, frequent opioid use in older adults—often in the treatment of nonspecific pain—has contributed to the rise in opioid overuse within this population. The use of GLP-1 RAs, due to their activity in the nucleus accumbens and related brain regions, has been associated with a reduction in opioid dependence, including oxycodone [51].

In a randomized controlled trial evaluating GLP-1 RAs for opioid use disorder (OUD), participants receiving low-dose liraglutide reported a 40% reduction in opioid craving over a 3-week period. However, over half of the study participants discontinued participation before completion, significantly limiting the study’s overall validity [5].

An observational study involving 33,000 patients with T2DM showed that treatment with semaglutide was associated with a nearly 50% lower risk of opioid overdose compared to other antidiabetic medications over a one-year follow-up period (HR ranging from 0.32 (95% CI, 0.12–0.89) to 0.58 (95% CI, 0.38–0.87)) [52].

A recent literature review of both preclinical and clinical studies further supports that GLP-1 RAs may reduce the risk of opioid overdose, opioid-seeking behaviors, and opioid self-administration. Multiple clinical trials aimed at evaluating their use in the treatment of opioid use disorder are currently underway, with results expected by 2027 [51].

#### 3.3.5. GLP-1 RAs on Mental Health—Depression and Suicide

Depressive disorders are among the most prevalent psychiatric conditions, characterized by diverse clinical presentations and a profound impact on psychosocial functioning. According to the World Health Organization (WHO), depression is one of the leading causes of global disability, both mental and physical.

Psychological pain is an integral component of depressive disorders and is strongly linked to an increased risk of suicidal ideation and suicidal behavior [53]. Individuals with depression exhibit increased sensitivity to both psychological and physical pain [54]. In recent years, there has been growing interest in the potential psychotropic effects of GLP-1 RAs on mental health.

Initially, pharmacovigilance reports involving liraglutide and semaglutide raised concerns about a possible association with increased suicidal ideation and self-injurious behavior. However, recently published clinical data indicate no causal relationship between GLP-1 RA use and suicidal behavior in individuals with psychiatric conditions [55].

A large-scale retrospective study conducted by Jianxing Zhou et al. on 93,431 clinical cases, including 204 involving suicidal and self-injurious behavior (SSIB), found no significant association between GLP-1 RA treatment and the occurrence of SSIB [56].

Furthermore, preclinical studies in animal models have demonstrated antidepressant-like effects of dulaglutide, including the reversal of depressive-like behaviors induced by chronic stress and inflammation [57].

A meta-analysis by Xinda Chen et al., including over 2000 participants across five randomized controlled trials and one prospective cohort study, showed that adults treated with GLP-1 RAs experienced significantly fewer depressive symptoms compared to those treated with other antidiabetic medications (SMD = −0.12, 95% CI [−0.21, −0.03], *p*_SMD_ < 0.01, *I*^2^ = 0%, *p*_Q_ = 0.52). These findings suggest that GLP-1 RAs may offer therapeutic benefit in alleviating depressive symptoms, particularly among patients treated for obesity [58,59].

However, both preclinical and early human studies suggest an anxiolytic potential of exenatide. The hypothesized mechanisms include modulation of serotonin and GABAergic neurotransmission, enhancement of neuroplasticity, and anti-inflammatory properties [60].

A recent 2025 systematic review and meta-analysis further strengthens these findings, reporting no significant association between GLP-1 RA use and increased risk of suicide or self-harm across both clinical trials and observational studies. Even though there is strong preclinical evidence, observational results are mixed, and clinical findings are still in the preliminary stage. Most of the evidence supporting GLP-1RAs comes from preclinical studies or studies involving populations with metabolic disorders [61].

Baseline severity of depression, the use of antidepressants, comorbid substance use, and pre-existing psychiatric diagnoses are strong confounding factors in observational studies. The results obtained so far may reflect confounding by indication and differential access to healthcare. Therefore, rigorous short- and long-term randomized controlled trials are needed to confirm the hypothesized role of GLP-1 RAs in the treatment and/or prevention of depressive symptoms and episodes in adults with major depressive disorder (MDD).

#### 3.3.6. GLP-1 RAs on Kidney Disorders

T2DM significantly increases the risk of developing cardiovascular diseases and diabetic nephropathy, which can progress to chronic kidney disease (CKD). Perkovic et al. demonstrated that semaglutide significantly reduces the risk of kidney function decline and cardiovascular death in patients with T2DM and CKD [9]. A randomized FLOW trial involving 3533 participants (median follow-up 3.4 years), showed that the risk of a primary-outcome event (major kidney disease events, kidney failure, ≥50% eGFR reduction from baseline, renal/cardiovascular death) was 24% lower in the semaglutide group than in the placebo group (331 vs. 410 first events; HR, 0.76; 95% CI, 0.66 to 0.88; *p* = 0.0003). Similar results were achieved for a composite of the kidney-specific components of the primary outcome (HR, 0.79; 95% CI, 0.66 to 0.94) and for death from cardiovascular causes (HR, 0.71; 95% CI, 0.56 to 0.89). The risk of major cardiovascular events was 18% lower (HR, 0.82; 95% CI, 0.68 to 0.98; *p* = 0.029). Furthermore, the risk of all-cause mortality was reduced by 20% (HR, 0.80; 95% CI, 0.67–0.95; *p* = 0.01). Regarding surrogate renal outcomes, semaglutide treatment resulted in a significantly slower decline in kidney function, with the mean annual eGFR slope being 1.16 mL/min/1.73 m^2^ less steep compared to placebo (*p* < 0.001) [9,62].

A recent 2025 meta-analysis of 24 randomized controlled trials (RCTs) confirmed that GLP-1 RAs positively influence renal surrogate parameters in patients with T2DM. The study reported a significant reduction in serum creatinine levels (WMD = −0.10, 95% CI: −0.19 to −0.01, *I*^2^ = 33%, *p*  <  0.05) and urinary albumin-to-creatinine ratio (UACR) (WMD: −1.01 mg/g, 95% CI:−1.68, −0.34, *I*^2^ = 15%, *p* < 0.05). Furthermore, the study underlines statistically significant elevation in eGFR (WMD = 0.54, 95% CI 0.19 to 0.90, *I*^2^ = 27%, *p* < 0.05) compared to control groups [63].

Clinicians studying the renal effects of GLP-1 RAs in patients with diabetes point to genetic evidence supporting the nephroprotective role of these agents in both diabetic nephropathy and IgA nephropathy. Inflammation plays a central role in the pathogenesis of IgA nephropathy, and the anti-inflammatory properties of GLP-1 RAs may form the basis of their therapeutic potential [64]. However, clinical trials are needed to confirm their efficacy and safety in this setting [65].

GLP-1 RAs also activate AMP-activated protein kinase (AMPK), a key enzyme regulating cellular energy metabolism, including cardiac muscle, likely contributing to their cardiovascular protective effects.

RCT meta-analyses have shown that GLP-1 RAs reduce the risk and progression of CKD. While the exact mechanism remains unclear, these effects are likely mediated by AMPK activation and testosterone suppression, also implicated in prostate cancer development.

#### 3.3.7. GLP-1 RAs in Obesity and Prostate Cancer

Studies show that prostate cancer (PCa) is the most common malignancy and the second leading cause of cancer-related death among men in the United States [66]. Insulin metabolism may be a key link between PCa, obesity, and metabolic disorders. One of the molecular factors identified in this signaling cascade is the FOXC2 (forkhead box C2) transcription factor, which may act as a connector between PCa, metabolic syndrome, and obesity. FOXC2 is known to suppress genes involved in insulin resistance while promoting the proliferation of prostate cancer cells [67].

The interaction between insulin resistance and PCa remains incompletely understood. Insulin signaling is thought to play an important role in the crosstalk between adipocytes and tumor cells, potentially influencing tumor proliferation and metabolic remodeling within the tumor microenvironment. Insulin resistance may promote PCa progression by altering adipocyte function and stimulating the release of bioactive mediators that support tumor growth [68].

The tumor microenvironment, particularly the surrounding adipose tissue, plays a key role in this bidirectional communication through the secretion of adipokines, cytokines, and growth factors, as well as through altered gene expression that may facilitate cancer progression [69]. In the context of PCa, adipocytes located in the periprostatic adipose tissue represent an important source of signaling molecules that can promote tumor proliferation, invasion, and metastasis. Among these mediators, decreased levels of adiponectin have been associated with an increased risk of PCa progression and more aggressive disease characteristics [70]. Additionally, adipose tissue–derived adipokines may further modulate tumor behavior, while hypoxic conditions within the tumor microenvironment may enhance the metastatic potential of PCa cells.

GLP-1RAs contribute to reductions in body weight and adipose tissue, which may lead to improvements in the adipokine profile. Liraglutide also decreases fasting serum leptin levels, resulting in a significant reduction in the leptin-to-adiponectin ratio [71].

FOXC2, an effector of the EGFR signaling pathway, plays a key role in energy metabolism, cell growth, and differentiation in obesity and prostate cancer. In obesity, elevated leptin and reduced adiponectin levels promote chronic low-grade inflammation via pro-inflammatory cytokines. When adipose tissue becomes insulin-resistant, EGFR and IGF-1 receptors are activated, triggering overexpression of pro-tumorigenic factors. Insulin and TNF-α are known to induce FOXC2 in 3T3-L1 adipocytes. Meanwhile, free IGF-1 and its binding proteins (IGFBP-1 and IGFBP-3) exert opposing antitumor effects [72].

Emerging evidence suggests a potential association between GLP-1 RA use and a lower risk of prostate cancer. In the LEADER trial, patients treated with liraglutide had a lower incidence of prostate cancer compared to placebo [73]. The randomized, double-blind LEADER trial, designed to assess cardiovascular risk, was not intended to evaluate the risk of developing cancer. However, a secondary outcome analysis revealed a lower incidence of prostate cancer in the liraglutide group (*n* = 26) compared to the placebo group (*n* = 47), with an HR of 0.54 (95% CI: 0.34–0.88). While these findings are hypothesis-generating and do not allow for definitive causal claims regarding a protective effect, they confirm the safety profile of liraglutide therapy concerning prostate malignancy in patients with T2DM [74]. Similarly, a large-scale cohort study involving 1.1 million individuals with obesity demonstrated a reduction in prostate cancer risk among users of GLP-1 RAs [75]. This association has also been noted for gastrointestinal, skin, breast, and hematopoietic malignancies, most of which are known to be obesity-related.

Moreover, GLP-1 RAs were found to significantly reduce all-cause mortality in cancer survivors [76]. Notably, in patients with prostate cancer, liraglutide may serve as an adjunct to standard treatments, potentially enhancing the effectiveness of radiotherapy, hormone therapy, and chemotherapy [77].

Although the direct or indirect role of IGF signaling in FOXC2 regulation and PCa development is not yet fully established, existing evidence supports the need for further studies to better understand these interactions. Lifestyle, diet, and genetic predispositions also play significant roles in the pathophysiology of metabolic disorders and PCa.

#### 3.3.8. GLP-1 RAs on Neuroprotection

Alzheimer’s disease (AD) is the most common neurodegenerative disorder and represents a significant global health challenge. Its progressive cognitive decline markedly reduces patients’ quality of life, ultimately leading to disability and premature death [78,79].

From an epidemiological and pathophysiological perspective, AD and T2DM share numerous similarities and are sometimes referred to as “sister diseases” [28,30,80]. T2DM increases the risk of developing AD, while neurodegenerative processes impair systemic glucose metabolism. Consequently, AD has been termed “type 3 diabetes” [30].

Given the underlying mechanisms and etiology of AD, there is growing interest in the potential neuroprotective effects of GLP-1 RAs [81]. This hypothesis is supported by studies from teams at Harbin Medical University and Huazhong University of Science and Technology, which demonstrated that GLP-1 RAs exert anti-inflammatory effects in the central nervous system and reduce microglial activation. They also decrease pathological deposition of β-amyloid and tau proteins, potentially by enhancing autophagy and clearance of damaged cellular structures. Additionally, GLP-1 RAs promote long-term synaptic potentiation and cognitive functions and reduce oxidative stress by lowering free radical production and increasing antioxidant enzyme activity [78].

These findings are corroborated by a large cohort study conducted by Siddeeque et al., which found that semaglutide use was associated with a 37% reduction in the risk of developing AD (RR = 0.627, 95% CI = 0.481–0.817, *p* < 0.001) and over 50% reduction in all-cause mortality (HR = 0.525, 95% CI = 0.493–0.558, *p* < 0.001). Furthermore, GLP-1 RAs reduced the risk of Lewy body dementia and vascular dementia by 41% (RR = 0.590, 95% CI = 0.462–0.753, *p* < 0.001) and 56% (RR = 0.438, 95% CI = 0.327–0.588, *p* < 0.001), respectively. This study analyzed data from 5,307,845 obese adult patients across 73 healthcare organizations in 17 countries, forming two cohorts of 102,935 patients each [82].

Regarding Parkinson’s disease (PD), available evidence remains inconsistent. In preclinical models, tirzepatide significantly reduced neuroinflammation and oxidative stress in PD mice, increased striatal dopamine levels, and promoted mitochondrial homeostasis [83]. More broadly, GLP-1 RAs have been shown to support the survival of dopaminergic neurons by inhibiting apoptosis and inflammatory pathways, as well as by restoring brain insulin signaling, thereby targeting metabolic dysfunction implicated in PD pathogenesis.

However, clinical evidence has not consistently confirmed these neuroprotective effects. In the cohort analysis by Siddeeque et al., a trend toward reduced PD risk was observed among GLP-1 RA users, but the association did not reach statistical significance [82]. Similarly, a systematic review and meta-analysis conducted by researchers from Universidade do Estado do Amazonas reported no significant improvement in any component of the MDS-UPDRS scale (I–IV) after 6 and 12 months of GLP-1 RA treatment. No meaningful changes in quality of life measured by PDQ-39 were observed, while treatment was associated with increased gastrointestinal adverse events and weight loss [84]. Consistent findings were reported in a recent meta-analysis evaluating key clinical outcomes in PD, which showed no significant improvements in MDS-UPDRS Parts II and IV [85].

In contrast, several large observational studies suggest a potential reduction in PD incidence associated with GLP-1 RA use. A nationwide cohort study conducted in Denmark included 33,462 patients initiating either GLP-1 RAs or dipeptidyl peptidase-4 inhibitors (DPP-4i) between 2007 and 2018, with follow-up until 2022. After 10 years of sustained treatment, GLP-1 RA users showed a lower risk of PD compared with matched DPP-4i users (HR 0.57; 95% CI 0.37–0.85), with similar trends observed when insulin was used as a comparator. During follow-up, 192 participants developed PD, including 93 cases occurring during sustained treatment exposure [86]. Comparable results were reported in a population-based cohort study using U.S. Medicare data (2016–2020), which included 89,074 adults aged ≥66 years with T2DM. In this analysis, initiation of GLP-1 RA therapy was associated with a 23% lower risk of PD compared with DPP-4 inhibitor use (HR 0.77; 95% CI 0.63–0.95), with lower crude incidence rates among GLP-1 RA users (2.85 vs. 3.92 cases per 1000 person-years) [87].

Taken together, these findings suggest that GLP-1 RAs may represent a promising therapeutic strategy that targets both central neurodegenerative mechanisms and systemic metabolic dysfunction associated with PD. Their potential to influence disease risk and possibly disease progression has generated considerable interest, particularly within the framework of personalized therapeutic approaches. Nevertheless, current clinical evidence regarding their impact on PD progression remains inconclusive. Some of the discrepancies may be partly explained by treatment-related adverse effects such as gastrointestinal symptoms and weight loss, which may influence functional outcome measures. Therefore, further well-designed prospective and randomized studies are required to clarify the potential neuroprotective role of GLP-1 RAs in PD [88].

Studies investigating GLP-1’s role in amyotrophic lateral sclerosis (ALS) pathogenesis and progression showed elevated GLP-1 levels in ALS patients compared to healthy controls, although the correlation with the ALS functional rating scale (ALSFRS-R) was insignificant [89].

Despite the neuroprotective effects of GLP-1 RAs by restoring neurite outgrowth, enhancing neurotrophic factors, and reinforcing the blood – brain barrier, the inconsistent results in different neurodegenerative disorders highlight the need for further research to clarify their potential therapeutic role.

Another important methodological issue in studies evaluating neurodegenerative outcomes is potential reverse causality. The early stages of neurodegenerative diseases, including AD and other types of dementia, may be associated with weight loss, metabolic changes, and changes in treatment regimens before formal diagnosis. These changes may occur many years before diagnosis. Additionally, they may influence prescribing patterns, including reducing the likelihood of initiating newer diabetes therapies such as GLP-1 RAs. As a result, observational studies based on large healthcare databases may misattribute the protective effect to exposure to GLP-1 RAs, when in fact the underlying neurodegenerative process may have preceded the initiation of treatment. This phenomenon could potentially exaggerate the apparent protective effect of GLP-1 Ras on neurodegenerative outcomes. Future pharmacoepidemiological studies should account for this limitation by employing methodological approaches such as delayed exposure analyses, which exclude drug exposure in the period immediately preceding diagnosis, thereby reducing the risk of reverse causality.

#### 3.3.9. GLP-1 RAs on Fatty Liver Disease

Metabolic dysfunction-associated steatotic liver disease (MASLD), characterized by excessive fat accumulation within hepatocytes, can contribute to the development of cirrhosis and liver failure [90,91]. GLP-1 RAs reduce liver fat content and liver enzyme levels, decrease inflammation, oxidative stress, and liver fibrosis, improve hepatic de novo lipogenesis, thereby enhancing liver function [92,93]. It is believed that weight loss may contribute to these effects, although the exact mechanisms remain unclear. Endogenous glucose-induced GLP-1 secretion is reduced in patients with MASLD and metabolic dysfunction-associated steatohepatitis (MASH), which may represent another starting point for research into MASLD pathophysiology [93,94].

One study compared exenatide at a dose of 10 μg twice daily to insulin boluses in 60 patients with obesity, T2DM, and MASLD with elevated liver enzymes. After 12 weeks, levels of alanine aminotransferase (ALT), aspartate aminotransferase (AST), and γ-glutamyltransferase were lower in the exenatide group compared to insulin [92]. In another randomized clinical trial, retatrutide administered in advanced MASLD eliminated 86% of liver fat in patients.

Clinical trials of GLP-1 RAs have demonstrated various potential benefits in patients with MASH and MASLD, including resolution of steatohepatitis, reduction in liver stiffness, and fibrosis progression. The impact of GLP-1 RAs on the spectrum of fibrosis severity in MASH represents a crucial area for future research. Currently, trials of retatrutide in individuals with diabetes and obesity or overweight (TRIUMPH-2, NCT05929079) are ongoing, with results expected in 2026. Studies on a long-acting triple agonist of GLP-1/glucagon/GIP, HM15211 (efocipegtrutide), are in phase 2 for MASH (NCT04505436), with outcomes anticipated in 2025 [92,93].

GLP-1 is also significantly related to metabolic dysfunction-associated steatohepatitis (MASH), characterized by liver inflammation and hepatocyte injury. MASH is currently recognized as a leading cause of the rising incidence of hepatocellular carcinoma (HCC) [82], although its molecular mechanisms are complex and multifactorial [84]. Furthermore, GLP-1 RAs have been shown to effectively reduce body weight and liver fat content in patients with MASH [92].

#### 3.3.10. GLP-1 RAs on Hepatocellular Carcinoma

Liver cancer ranks as the sixth most common cancer worldwide. Hepatocellular carcinoma (HCC) accounts for approximately 72% of primary liver cancers [95]. HCC primarily develops in patients with post-inflammatory liver cirrhosis (80–90%), associated with HBV or HCV infections and their coexistence. Modifiable risk factors such as T2DM, obesity, alcohol and tobacco abuse, metabolic dysfunction-associated steatotic liver disease (MASLD), and metabolic dysfunction-associated steatohepatitis (MASH) also influence HCC development.

A cohort study of 1,890,020 patients with T2DM demonstrated that GLP-1 RAs were associated with a lower risk of HCC compared to other antidiabetic medications (insulin, metformin, DPP-4 inhibitors, SGLT2 inhibitors, sulfonylureas, and thiazolidinediones). GLP-1 RAs were also linked to reduced risk of liver function decompensation compared to other antidiabetics in diabetic patients. They were effective across different stages of MASLD, MASH, liver fibrosis, and cirrhosis, with the greatest efficacy observed in patients without prior liver disease. GLP-1 RAs indirectly reduce HCC risk by influencing the reward system and addiction to smoking and alcohol. Moreover, combination therapies including GLP-1 RAs offer additional benefits over monotherapies [95].

Well-established metabolic effects of GLP-1 RAs appear to be accompanied by epigenetic modulatory properties, such as inhibiting lactate-mediated histone lactylation in fibrotic contexts, presenting a novel strategy to inhibit progression from chronic liver injury through fibrosis to HCC [96].

#### 3.3.11. GLP-1 RAs on Sleep Apnea

Obstructive sleep apnea (OSA) is a component of metabolic syndrome. The randomized SURMOUNT-OSA trials (Trial 1 and 2), including patients with obstructive sleep apnea (OSA), demonstrated that tirzepatide treatment for one year resulted in significant clinical improvements. Tirzepatide led to a body weight reduction of approximately 20%, with an estimated treatment difference (ETD) of −16.1% (95% CI −18.0, −14.2) to −17.3% (−19.3, −15.3) compared to placebo. Most importantly, the treatment resulted in a nearly 60% decrease in apnea episodes, achieving an ETD in the Apnea-Hypopnea Index (AHI) of −47.7% (−65.8, −29.6) to −56.2% (−73.7, −38.7). However, it remains unclear whether the decrease in apnea episodes during sleep was dependent on weight loss only. Exploration of the relationship between the AHI improvement degree and the weight reduction magnitude would be important [97,98]. In the SCALE study focusing on sleep apnea in obese individuals with moderate to severe OSA who were unwilling or unable to use continuous positive airway pressure (CPAP), liraglutide was associated with a significantly greater reduction in apnea-hypopnea index and hypoxemia compared to placebo, alongside weight loss and improvements in systolic blood pressure and HbA1c levels [93].

#### 3.3.12. GLP-1 RAs on Arthritis

Osteoarthritis (OA) is a leading cause of disability, affecting over 300 million people worldwide. OA commonly involves the knees, hands, hips, and spine. Risk factors for knee and hip OA include age, sex, obesity, cardiometabolic factors, injuries, physical activity, and anatomical abnormalities. OA is suspected to be a component of metabolic syndrome [99]. The trial, including 39,394 patients with obesity using anti-obesity medications (AOMs) (23,933 semaglutide, 12,854 tirzepatide, 2607 liraglutide) and 72,405 without AOM use, demonstrated that the adjusted osteoarthritis risk was 27% lower in AOM users than in non-users (HR = 0.73, 95% CI (0.67–0.79), *p* < 0.01). Randomized controlled trials have shown that overweight/obese individuals who lost an average of 14% of their body weight during semaglutide therapy experienced significant improvement in knee osteoarthritis pain, likely related to both weight loss and the anti-inflammatory properties of the drug. These results encourage consideration of GLP-1 RAs as potential treatment in patients requiring joint replacement surgery; however, further research is needed [100,101]. The discovery of anti-inflammatory, immunoregulatory, and differentiating effects of GLP-1 RAs at the joint tissue and cellular level raises the hypothesis of their potential use in OA treatment [99].

#### 3.3.13. GLP-1 RAs on PCOS, Libido, Fertility

Polycystic ovary syndrome (PCOS) is the most common cause of anovulatory infertility, affecting approximately 20% of women of reproductive age (according to Rotterdam criteria). Over half of women with PCOS struggle with overweight or obesity, which negatively impacts implantation rates, pregnancy numbers, and increases miscarriage risk [102].

GLP-1 RAs demonstrate beneficial effects in women with PCOS, who often present with comorbidities such as obesity, insulin resistance, glucose metabolism disorders, dyslipidemia, hypertension, or metabolic syndrome, all increasing diabetes risk. This therapy helps regulate appetite, improve glycemic control, induce weight loss, and gradually reduce metabolic disturbances that indirectly affect hormonal balance [44,103,104,105]. In patients with the so-called metabolic phenotype, an additional benefit is the reduced cardiovascular risk.

In an open-label, prospective, randomized clinical trial conducted over 12 months (September 2014–May 2015) with 28 obese women diagnosed with PCOS per Rotterdam criteria, preconception low-dose liraglutide combined with metformin was more effective than metformin alone in increasing pregnancy rates in IVF procedures, despite similar weight loss effects in both groups [102].

Studies suggest that GLP-1 RAs may support fertility by enhancing the preovulatory luteinizing hormone surge, leading to increased estrogen levels and restoration of normal menstrual cycles. Especially in women with PCOS, many begin ovulating again after significant visceral fat loss during tirzepatide or semaglutide therapy [93,106,107].

A literature review by Abdalla et al. showed that exenatide, both as monotherapy and combined with metformin, improves menstrual regularity and ovulation rates in overweight or obese women with PCOS, directly increasing fertility [108]. In a study by Karen Elkind-Hirsch et al. involving 60 obese women with oligo-ovulation and PCOS (70% completed the study), combination therapy with exenatide and metformin resulted in greater improvement in menstrual cyclicity, endogenous ovulatory function, body weight, waist circumference, insulin resistance, and hyperandrogenism compared to monotherapy [109].

Notably, women on GLP-1 RAs experience decreased testosterone levels—a key hormone for maintaining normal libido, whose excess can impair fertility. However, the impact of GLP-1 Ras on libido remains controversial; some patients report decreased libido during therapy, while overweight or obese individuals who lost weight report improved self-esteem, vitality, and sexual activity [110].

In men, obesity significantly affects the hypothalamic-pituitary-gonadal axis, resulting in decreased testosterone levels and diminished libido. Although data are limited, a possible role for GLP-1 Ras in treating functional hypogonadism related to overweight and obesity has been suggested [111]. In a retrospective observational study on 43 men, liraglutide proved superior to testosterone supplementation in obese men with functional hypogonadism [112].

In diabetic mice, liraglutide was shown to improve erectile function by relaxing corpus cavernosum smooth muscle cells and alleviating hypogonadism symptoms post-orchidectomy, while exenatide improved sperm quality in obese mice [113,114]. GLP-1 receptors have also been found in human testes, and some studies confirm improvements in erectile dysfunction in obese diabetic men treated with GLP-1 RAs. Positive effects on semen quality were suggested in a study by La Vignera et al., but it remains unclear whether these were due to weight loss or direct drug effects [112].

## 4. Safety of GLP-1 RAs

Although GLP-1 RAs are generally considered to have a favorable safety profile, their use is associated with adverse effects that may affect treatment tolerance and adherence to therapeutic recommendations. Gastrointestinal symptoms, including nausea, vomiting, diarrhea, and constipation, are the most commonly reported adverse events and often occur during the dose escalation phase of therapy. In clinical trials, these events may lead to dose adjustment or discontinuation of treatment in some patients [115].

In addition to typical gastrointestinal symptoms, some studies have reported associations with biliary tract disorders, including gallstones, as well as other gastrointestinal complications, although the absolute risk appears to be relatively low [116].

## 5. Risk of Bias and Confounding in Cited Studies

Several sources of bias should be considered when interpreting findings from observational studies evaluating GLP-1 RAs. In routine clinical practice, patients receiving GLP-1 RAs may differ systematically from those treated with other glucose-lowering therapies. Due to their relatively recent introduction and higher cost in some healthcare systems, GLP-1 RAs may be prescribed more frequently to younger individuals or patients with higher socioeconomic status. Such patients may also be more engaged with healthcare services and more likely to adopt lifestyle modifications, which could contribute to improved outcomes independent of pharmacological effects. 

Another important methodological concern in pharmacoepidemiological studies using large healthcare databases is immortal time bias. It occurs when a period of follow-up during which the outcome cannot occur is incorrectly classified as exposed time. In studies evaluating GLP-1 RAs, this may arise when patients are classified as exposed based on a prescription recorded after cohort entry, which can artificially prolong event-free follow-up in the treatment group. As a result, the treatment may appear to confer a protective effect even in the absence of a causal relationship. Outcomes discussed in this review—such as opioid overdose, neurodegenerative disorders, and cancer incidence—may be particularly sensitive to this bias when exposure is not treated as time-dependent.

Future observational studies should account for these limitations by adjusting for socioeconomic and clinical differences between treatment groups and by using appropriate pharmacoepidemiological methods, including time-dependent exposure models, new-user study designs, and sensitivity analyses that reduce misclassification of exposure time.

## 6. Strengths and Limitations of the Review

### 6.1. Limitations

#### 6.1.1. Difficulty in Distinguishing Direct Effects from Those Secondary to Weight Loss

One of the major challenges in interpreting data on the pleiotropic effects of GLP-1RAs is the difficulty in distinguishing direct pharmacological effects from those secondary to weight reduction. In several conditions, including obstructive sleep apnea, osteoarthritis, non-alcoholic fatty liver disease, fertility disorders, and depressive symptoms, observed clinical improvements may largely result from weight loss.

At the same time, it should be emphasized that weight reduction itself represents an important therapeutic mechanism that may contribute to lowering the risk and severity of many obesity-related comorbidities. As emphasized by Wojciech Bik, Vice-President of the Polish Society of Neuroendocrinology and Head of the Department of Clinical Neuroendocrinology at the Centre of Postgraduate Medical Education in Warsaw, obesity has been described as the “mother of all diseases,” highlighting its central role in the pathogenesis of numerous chronic conditions.

An additional challenge in separating these mechanisms arises from the fact that adipose tissue functions as an active endocrine organ that produces numerous adipokines and inflammatory mediators affecting multiple physiological systems. Consequently, some of the observed clinical outcomes may result both from the direct pharmacological action of GLP-1 RAs and from secondary metabolic changes associated with weight reduction.

In cases where clear evidence supporting a direct mechanism of action is lacking, the reported associations should be interpreted as hypotheses requiring further verification in studies specifically designed to evaluate these mechanisms.

#### 6.1.2. Limitations Related to the Nature of Available Studies

A substantial proportion of the evidence regarding the pleiotropic effects of GLP-1RAs originates from secondary analyses of clinical trials, observational studies, follow-up analyses, or preclinical investigations. In many cases, the analyzed outcomes were not the primary endpoints of the original studies.

Therefore, some of the observed associations should be considered exploratory and require confirmation in studies specifically designed to evaluate these clinical effects.

#### 6.1.3. Risk of Confounding in Real-World Evidence Studies

As relatively new therapeutic agents, GLP-1 RAs remain less accessible in many countries compared to other drugs used to treat metabolic or cardiovascular diseases.

Consequently, the population of patients receiving GLP-1RAs may be characterized by higher socioeconomic status, better access to healthcare, and greater health awareness. These factors may influence lifestyle, frequency of diagnostic testing, and earlier detection of diseases, which in turn may affect the observed clinical outcomes.

#### 6.1.4. Lack of a Clear Class Effect

Although GLP-1 analogues and newer dual or triple incretin receptor agonists share similar metabolic mechanisms of action, available evidence suggests that a uniform class effect may not exist for all pleiotropic outcomes. Individual molecules may differ in their pharmacodynamic profiles, receptor affinities, pharmacokinetics, and metabolic effects. Therefore, findings obtained for a specific drug cannot always be directly extrapolated to the entire therapeutic class.

#### 6.1.5. Limited Duration of Follow-Up

In many studies, the duration of follow-up was relatively short, which limits the ability to assess the long-term effects of the treatment on the development or progression of certain chronic diseases.

#### 6.1.6. Heterogeneity of Studied Populations

The studies included populations differing in treatment indications (e.g., T2DM or obesity), comorbidities, and concomitant therapies. This heterogeneity may complicate direct comparisons between studies and limit the generalizability of some findings.

### 6.2. Strengths

#### 6.2.1. Broad Scope of Data Analyzed

One of the major strengths of this review is the inclusion of studies in which potential pleiotropic effects of GLP-1 RAs were not the primary endpoints. In many cases, these associations were identified through secondary analyses or clinical observations among patients treated with GLP-1RAs. This approach allows for the identification of potentially relevant clinical effects that may guide the design of future targeted studies.

#### 6.2.2. Inclusion of Data from Diverse Populations

The review includes studies conducted in different countries and populations with diverse demographic and clinical characteristics, thus increasing the generalizability of the findings and allowing for the evaluation of GLP-1 RAs across different healthcare systems and patient populations.

#### 6.2.3. Interdisciplinary Scope of the Analysis

The review encompasses a wide range of disease entities representing multiple medical disciplines, including metabolic, cardiovascular, hepatological, and neurological disorders. Such an interdisciplinary approach enables a comprehensive overview of the pleiotropic actions of GLP-1 RAs and highlights the potential of this drug class beyond its original therapeutic indications.

#### 6.2.4. Scientific Independence

This work was conducted without financial support from the pharmaceutical industry, which reduces the potential risk of conflicts of interest and supports its independent scientific character.

#### 6.2.5. Timeliness of the Topic

The pleiotropic effects of GLP-1 RAs represent a rapidly evolving area of clinical research. Our review provides a structured synthesis of the currently available evidence regarding their potential extra-metabolic effects and identifies areas that require further investigation.

#### 6.2.6. Integration of Evidence from Different Study Types

The review integrates findings from clinical trials, observational studies, and preclinical research, allowing for a more comprehensive understanding of the potential mechanisms of action of GLP-1 RAs.

#### 6.2.7. Identification of Future Research Directions

The conducted review highlights areas where the available evidence remains limited or inconsistent, thereby identifying potential directions for future clinical and mechanistic studies.

## 7. Conclusions

**Figure 1:**
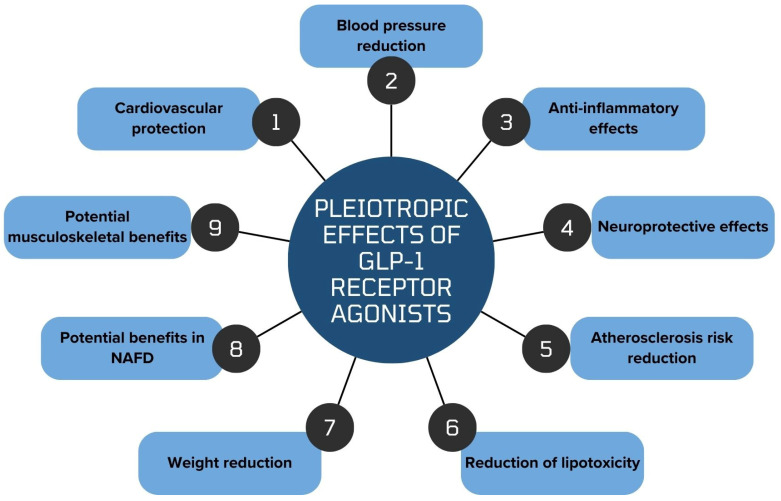
Pleiotropic effects associated with GLP-1 receptor agonists across multiple physiological systems. Abbreviations: GLP-1, glucagon-like peptide-1; MAFLD, metabolic dysfunction-associated fatty liver disease.

GLP-1 RAs demonstrate multifaceted associations with potential therapeutic effects, providing clinical benefits primarily in the management of T2DM and obesity, as supported by randomized controlled trials. Observational and preclinical studies further suggest possible benefits in cardiovascular diseases, neurodegenerative disorders, and certain cancers, although causality has not been established. Additionally, these agents may be associated with improvements in conditions such as sleep apnea, musculoskeletal disorders, PCOS, prostate enlargement, liver dysfunction, and even substance use and mental health, based largely on preclinical evidence or database analyses. GLP-1 RAs are generally characterized by a favorable safety profile, with adverse effects typically mild and rarely leading to treatment discontinuation. While current evidence is promising, further large-scale, well-designed clinical trials are required to clarify the extent of these potential effects and their clinical relevance. Overall, based on available data, GLP-1 RAs represent a promising therapeutic avenue with potential implications across multiple medical fields (Figure 1).

## Figures and Tables

**Table 1:** Literature search strategy and eligibility criteria.

Domain	Description
Databases searched	PubMed, Scopus, Google Scholar
Timeframe	2014–March 2026
Search terms	“GLP-1 receptor agonists”, “semaglutide”, “liraglutide”, “tirzepatide”, “GLP-1 analogues”, “neurological”, “mental health”, “nicotine”, “alcohol”, “addiction”, “prostate”, “cancer”, “sleep apnea”, “polycystic ovary syndrome (PCOS)”, “libido”, “fertility”, “neuroprotection”
Study types included	Randomized controlled trials, cohort studies, observational studies, mechanistic studies
Study types excluded	Case reports, conference abstracts, non-English articles
Study selection approach	Studies were selected based on relevance to the discussed clinical domains and methodological quality

Notes: Research on the pleiotropic effects of GLP-1 receptor agonists has expanded substantially over the past decade, particularly after 2014, when interest in their extra-glycemic effects significantly increased. Reference lists of relevant articles and reviews were also screened to identify additional eligible publications.

**Table 2:** Primary, evidence-based pharmacological effects of GLP-1 RAs.

GLP-1 RA	Indications	Impact on Disease—Confirmed in Studies	Research	Source
Semaglutide (subcutaneous)	T2DM; reduce the risk of CV events in high-risk patients; obesity	reduce the rate of eGFR decline	SUSTAIN 6 and PIONEER 6 trials, STEP 4 trial, STEP TEENS trial, SELECT trial, FLOW trial	[9,10,11,12,13,14]
Semaglutide (oral)	T2DM	reduce major adverse cardiovascular events	PIONEER 6 and SOUL trials	[15,16]
Liraglutide	T2DM; obesity; reduce the risk of CV events in high-risk patients		LEADER trial	[10,17]
Dulaglutide	T2DM; reduce the risk of CV events in high-risk patients	reduce the rate of eGFR decline	REWIND trial	[10,18]
Exenatide	T2DM	reduce major adverse cardiovascular events—not significantly	EXSCEL trial	[19,20]
Lixisenatide	T2DM	weight loss	ELIXA trial	[21,22,23]
Albiglutide	T2DM	reduce major adverse cardiovascular events	HARMONY trial	[24]
Tirzepatide	T2DM; obesity		SURPASS and SURMOUNT trials	[25,26]

## Data Availability

Publicly available datasets were analyzed in this study.

## References

[1] Parkes D.G., Mace K.F., Trautmann M.E. (2013). Discovery and development of exenatide: The first antidiabetic agent to leverage the multiple benefits of the incretin hormone, GLP-1. Expert Opin. Drug Discov..

[2] Knop F.K., Brønden A., Vilsbøll T. (2017). Exenatide: Pharmacokinetics, clinical use, and future directions. Expert Opin. Pharmacother..

[3] Nauck M.A., Quast D.R., Wefers J., Meier J.J. (2021). GLP-1 receptor agonists in the treatment of type 2 diabetes—State-of-the-art. Mol. Metab..

[4] Yao Y.X., Tang C., Si F.L., Lv J.C., Shi S.F., Zhou X.J., Liu L.J., Zhang H. (2025). Glucagon-like peptide-1 receptor agonists, inflammation, and kidney diseases: Evidence from Mendelian randomization. Ren. Fail..

[5] O’Keefe J.H., Franco W.G., O’Keefe E.L. (2025). Anti-consumption agents: Tirzepatide and semaglutide for treating obesity-related diseases and addictions, and improving life expectancy. Prog. Cardiovasc. Dis..

[6] Deanfield J., Verma S., Scirica B.M., Kahn S.E., Emerson S.S., Ryan D., Lingvay I., Colhoun H.M., Plutzky J., Kosiborod M.N. (2024). Semaglutide and cardiovascular outcomes in patients with obesity and prevalent heart failure: A prespecified analysis of the SELECT trial. Lancet.

[7] Borlaug B.A., Zile M.R., Kramer C.M., Baum S.J., Hurt K., Litwin S.E., Murakami M., Ou Y., Upadhyay N., Packer M. (2025). Effects of tirzepatide on circulatory overload and end-organ damage in heart failure with preserved ejection fraction and obesity: A secondary analysis of the SUMMIT trial. Nat. Med..

[8] Li Q.X., Gao H., Guo Y.X., Wang B.Y., Hua R., Gao L., Shang H.W., Lu X., Xu J.D. (2021). GLP-1 and Underlying Beneficial Actions in Alzheimer’s Disease, Hypertension, and NASH. Front. Endocrinol..

[9] Perkovic V., Tuttle K.R., Rossing P., Mahaffey K.W., Mann J.F.E., Bakris G., Baeres F.M.M., Idorn T., Bosch-Traberg H., Lausvig N.L. (2024). Effects of Semaglutide on Chronic Kidney Disease in Patients with Type 2 Diabetes. N. Engl. J. Med..

[10] Nauck M.A., Quast D.R. (2021). Cardiovascular Safety and Benefits of Semaglutide in Patients With Type 2 Diabetes: Findings From SUSTAIN 6 and PIONEER 6. Front. Endocrinol..

[11] Rubino D., Abrahamsson N., Davies M., Hesse D., Greenway F.L., Jensen C., Lingvay I., Mosenzon O., Rosenstock J., Rubio M.A. (2021). Effect of Continued Weekly Subcutaneous Semaglutide vs Placebo on Weight Loss Maintenance in Adults With Overweight or Obesity: The STEP 4 Randomized Clinical Trial. JAMA.

[12] Weghuber D., Barrett T., Barrientos-Pérez M., Gies I., Hesse D., Jeppesen O.K., Kelly A.S., Mastrandrea L.D., Sørrig R., Arslanian S. (2022). Once-Weekly Semaglutide in Adolescents with Obesity. N. Engl. J. Med..

[13] Tuttle K.R., Bosch-Traberg H., Cherney D.Z.I., Hadjadj S., Lawson J., Mosenzon O., Rasmussen S., Bain S.C. (2023). Post hoc analysis of SUSTAIN 6 and PIONEER 6 trials suggests that people with type 2 diabetes at high cardiovascular risk treated with semaglutide experience more stable kidney function compared with placebo. Kidney Int..

[14] Ryan D.H., Lingvay I., Deanfield J., Kahn S.E., Barros E., Burguera B., Colhoun H.M., Cercato C., Dicker D., Horn D.B. (2024). Long-term weight loss effects of semaglutide in obesity without diabetes in the SELECT trial. Nat. Med..

[15] Husain M., Birkenfeld A.L., Donsmark M., Dungan K., Eliaschewitz F.G., Franco D.R., Jeppesen O.K., Lingvay I., Mosenzon O., Pedersen S.D. (2019). Oral Semaglutide and Cardiovascular Outcomes in Patients with Type 2 Diabetes. N. Engl. J. Med..

[16] Marx N., Deanfield J.E., Mann J.F.E., Arechavaleta R., Bain S.C., Bajaj H.S., Tanggaard K.B., Birkenfeld A.L., Buse J.B., Davicevic-Elez Z. (2025). Oral Semaglutide and Cardiovascular Outcomes in People With Type 2 Diabetes, According to SGLT2i Use: Prespecified Analyses of the SOUL Randomized Trial. Circulation.

[17] Marso S.P., Daniels G.H., Brown-Frandsen K., Kristensen P., Mann J.F.E., Nauck M.A., Nissen S.E., Pocock S., Poulter N.R., Ravn L.S. (2016). Liraglutide and Cardiovascular Outcomes in Type 2 Diabetes. N. Engl. J. Med..

[18] Botros F.T., Gerstein H.C., Malik R., Nicolay C., Hoover A., Turfanda I., Colhoun H.M., Shaw J.E. (2023). Dulaglutide and Kidney Function–Related Outcomes in Type 2 Diabetes: A REWIND Post Hoc Analysis. Diabetes Care.

[19] Holman R.R., Bethel M.A., Mentz R.J., Thompson V.P., Lokhnygina Y., Buse J.B., Chan J.C., Choi J., Gustavson S.M., Iqbal N. (2017). Effects of Once-Weekly Exenatide on Cardiovascular Outcomes in Type 2 Diabetes. N. Engl. J. Med..

[20] Neves J.S., Leite A.R., Mentz R.J., Holman R.R., Zannad F., Butler J., Packer M., Ferreira J.P. (2025). Cardiovascular Outcomes with Exenatide in Type 2 Diabetes According to Ejection Fraction: The EXSCEL Trial. Eur. J. Heart Fail..

[21] Sheng L., Deng M., Li X., Wan H., Lei C., Prabahar K., Hernández-Wolters B., Kord-Varkaneh H. (2024). The effect of subcutaneous Lixisenatide on weight loss in patients with type 2 Diabetes Mellitus: Systematic review and Meta-Analysis of randomized controlled trials. Diabetes Res. Clin. Pract..

[22] Fountoulakis N., Pavlou P., Stathi D., Goubar A., Corcillo A., Flaquer M., Ayis S., Gnudi L., Karalliedde J. (2026). Effect of lixisenatide on arterial stiffness in people with type 2 diabetes and kidney disease: Results of a randomised controlled trial. Diabetes Obes. Metab..

[23] Gerstein H.C., Wolsk E., Claggett B., Diaz R., Dickstein K., Hess S., Køber L., Maggioni A.P., McMurray J.J.V., Probstfield J.L. (2023). Effect of lixisenatide on natriuretic peptides in people with type 2 diabetes and recent acute coronary syndrome: The ELIXA trial. Diabetes Obes. Metab..

[24] Krychtiuk K.A., Marquis-Gravel G., Murphy S., Alexander K.P., Chiswell K., Green J.B., Leiter L.A., Lopes R.D., Prato S.D., Jones W.S. (2024). Effects of albiglutide on myocardial infarction and ischaemic heart disease outcomes in patients with type 2 diabetes and cardiovascular disease in the Harmony Outcomes trial. Eur. Heart J.—Cardiovasc. Pharmacother..

[25] Rosenstock J., Frías J.P., Rodbard H.W., Tofé S., Sears E., Huh R., Landó L.F., Patel H. (2023). Tirzepatide vs Insulin Lispro Added to Basal Insulin in Type 2 Diabetes: The SURPASS-6 Randomized Clinical Trial. JAMA.

[26] Horn D.B., Kahan S., Batterham R.L., Cao D., Lee C.J., Murphy M., Gonsahn-Bollie S., Chigutsa F., Stefanski A., Dunn J.P. (2025). Time to weight plateau with tirzepatide treatment in the SURMOUNT -1 and SURMOUNT -4 clinical trials. Clin. Obes..

[27] Hendershot C.S., Bremmer M.P., Paladino M.B., Kostantinis G., Gilmore T.A., Sullivan N.R., Tow A.C., Dermody S.S., Prince M.A., Jordan R. (2025). Once-Weekly Semaglutide in Adults With Alcohol Use Disorder: A Randomized Clinical Trial. JAMA Psychiatry.

[28] Klausen M.K., Thomsen M., Wortwein G., Fink-Jensen A. (2022). The role of glucagon-like peptide 1 (GLP-1) in addictive disorders. Br. J. Pharmacol..

[29] Patel S., Blaney H., Nassar S., Singal A.K. (2025). GLP-1 receptor agonists and alcohol use disorder: A systematic review. Alcohol. Alcohol..

[30] Schooling C.M., Yang G., Soliman G.A., Leung G.M. (2025). A Hypothesis That Glucagon-like Peptide-1 Receptor Agonists Exert Immediate and Multifaceted Effects by Activating Adenosine Monophosphate-Activate Protein Kinase (AMPK). Life.

[31] Jerlhag E. (2018). GLP-1 signaling and alcohol-mediated behaviors; preclinical and clinical evidence. Neuropharmacology.

[32] Jerlhag E. (2025). GLP-1 Receptor Agonists: Promising Therapeutic Targets for Alcohol Use Disorder. Endocrinology.

[33] Wang W., Volkow N.D., Berger N.A., Davis P.B., Kaelber D.C., Xu R. (2024). Associations of semaglutide with incidence and recurrence of alcohol use disorder in real-world population. Nat. Commun..

[34] Nasrollahizadeh A., Kheiri G., Javankiani S., Kheiri S., Hamzavi S.F., Karimi M., Amini-Salehi E., Karimi M.A. (2026). Repurposing GLP-1 receptor agonists for alcohol use disorder: A systematic review and meta-analysis. Diabetol. Metab. Syndr..

[35] Srinivasan N.M., Farokhnia M., Farinelli L.A., Ferrulli A., Leggio L. (2025). GLP-1 Therapeutics and Their Emerging Role in Alcohol and Substance Use Disorders: An Endocrinology Primer. J. Endocr. Soc..

[36] Lee S., Li M., Le G.H., Teopiz K.M., Vinberg M., Ho R., Au H.C.T., Wong S., Valentino K., Kwan A.T.H. (2024). Glucagon-like peptide-1 receptor agonists (GLP-1RAs) as treatment for nicotine cessation in psychiatric populations: A systematic review. Ann. Gen. Psychiatry.

[37] Tuesta L.M., Chen Z., Duncan A., Fowler C.D., Ishikawa M., Lee B.R., Liu X.A., Lu Q., Cameron M., Hayes M.R. (2017). GLP-1 acts on habenular avoidance circuits to control nicotine intake. Nat. Neurosci..

[38] Wang W., Volkow N.D., Berger N.A., Davis P.B., Kaelber D.C., Xu R. (2024). Association of Semaglutide With Tobacco Use Disorder in Patients With Type 2 Diabetes: Target Trial Emulation Using Real-World Data. Ann. Intern. Med..

[39] Martinelli S., Mazzotta A., Longaroni M., Petrucciani N. (2024). Potential role of glucagon-like peptide-1 (GLP-1) receptor agonists in substance use disorder: A systematic review of randomized trials. Drug Alcohol. Depend..

[40] Hernandez N.S., Ige K.Y., Mietlicki-Baase E.G., Molina-Castro G.C., Turner C.A., Hayes M.R., Schmidt J.D. (2018). Glucagon-like peptide-1 receptor activation in the ventral tegmental area attenuates cocaine seeking in rats. Neuropsychopharmacology.

[41] Mugari I. (2024). The Emerging Trends and Response to Drug and Substance Abuse among the Youth in Zimbabwe. Soc. Sci..

[42] Al Khouri I., Iannaccone B., Fulajtarova M. (2024). Trends in Substance Use Among Young People in the EU: A Secondary Analysis of Available Data. Addictology.

[43] Stachelska A. (2025). Aktualne tendencje przestępczość narkotykowej oraz zażywania narkotyków wśród nieletnich w Polsce. Prawo Więź.

[44] Ceceli A.O., Bradberry C.W., Goldstein R.Z. (2022). The neurobiology of drug addiction: Cross-species insights into the dysfunction and recovery of the prefrontal cortex. Neuropsychopharmacology.

[45] Beheshti I. (2023). Cocaine Destroys Gray Matter Brain Cells and Accelerates Brain Aging. Biology.

[46] Egecioglu E., Engel J.A., Jerlhag E. (2013). The Glucagon-Like Peptide 1 Analogue, Exendin-4, Attenuates the Rewarding Properties of Psychostimulant Drugs in Mice. PLoS ONE.

[47] Chaves Filho A.J.M., Cunha N.L., De Souza A.G., Soares M.V.R., Jucá P.M., De Queiroz T., Oliveira J.V.S., Valvassori S.S., Barichello T., Quevedo J. (2020). The GLP-1 receptor agonist liraglutide reverses mania-like alterations and memory deficits induced by D-amphetamine and augments lithium effects in mice: Relevance for bipolar disorder. Prog. Neuropsychopharmacol. Biol. Psychiatry.

[48] Wang W., Volkow N.D., Berger N.A., Davis P.B., Kaelber D.C., Xu R. (2024). Association of semaglutide with reduced incidence and relapse of cannabis use disorder in real-world populations: A retrospective cohort study. Mol. Psychiatry.

[49] Arillotta D., Floresta G., Papanti Pelletier G.D., Guirguis A., Corkery J.M., Martinotti G., Schifano F. (2024). Exploring the Potential Impact of GLP-1 Receptor Agonists on Substance Use, Compulsive Behavior, and Libido: Insights from Social Media Using a Mixed-Methods Approach. Brain Sci..

[50] Au H.C.T., Lam P.H., Kabir F., Huang C.L., Dri C.E., Le G.H., Kwan A.T.H., Wong S., Teopiz K.M., McIntyre R.S. (2025). Glucagon-like peptide-1 receptor agonists for the treatment of opioid use disorders: A systematic review. Acta Neuropsychiatr..

[51] Qeadan F., McCunn A., Tingey B. (2025). The association between glucose-dependent insulinotropic polypeptide and/or glucagon-like peptide-1 receptor agonist prescriptions and substance-related outcomes in patients with opioid and alcohol use disorders: A real-world data analysis. Addiction.

[52] Wang W., Volkow N.D., Wang Q., Berger N.A., Davis P.B., Kaelber D.C., Xu R. (2024). Semaglutide and Opioid Overdose Risk in Patients With Type 2 Diabetes and Opioid Use Disorder. JAMA Netw. Open.

[53] Monroe S.M., Harkness K.L. (2022). Major Depression and Its Recurrences: Life Course Matters. Annu. Rev. Clin. Psychol..

[54] Conejero I., Olié E., Calati R., Ducasse D., Courtet P. (2018). Psychological Pain, Depression, and Suicide: Recent Evidences and Future Directions. Curr. Psychiatry Rep..

[55] McIntyre R.S. (2024). Glucagon-like peptide-1 receptor agonists (GLP-1 RAs) and suicidality: What do we know and future vistas. Expert Opin. Drug Saf..

[56] Zhou J., Zheng Y., Xu B., Long S., Zhu Le Liu Y., Li C., Zhang Y., Liu M., Wu X. (2024). Exploration of the potential association between GLP-1 receptor agonists and suicidal or self-injurious behaviors: A pharmacovigilance study based on the FDA Adverse Event Reporting System database. BMC Med..

[57] Darwish A.B., El Sayed N.S., Salama A.A.A., Saad M.A. (2023). Dulaglutide impedes depressive-like behavior persuaded by chronic social defeat stress model in male C57BL/6 mice: Implications on GLP-1R and cAMP/PKA signaling pathway in the hippocampus. Life Sci..

[58] Chen X., Zhao P., Wang W., Guo L., Pan Q. (2024). The Antidepressant Effects of GLP-1 Receptor Agonists: A Systematic Review and Meta-Analysis. Am. J. Geriatr. Psychiatry.

[59] Himmerich H. (2025). Glucagon-like-peptide-1-Rezeptoragonisten: Eine neue pharmakologische Behandlungsoption für psychiatrische Erkrankungen?. Nervenarzt.

[60] Komsuoglu Celikyurt I., Mutlu O., Ulak G., Uyar E., Bektaş E., Yildiz Akar F., Erden F., Tarkun I. (2014). Exenatide Treatment Exerts Anxiolytic- and Antidepressant-Like Effects and Reverses Neuropathy in a Mouse Model of Type-2 Diabetes. Med. Sci. Monit. Basic Res..

[61] Li S., Sabbah S.G., Kwan A.T.H., McIntyre R.S. (2025). Repurposing glucagon-like peptide-1 (GLP-1) receptor agonists for the treatment of depression: A systematic review of preclinical, observational and clinical investigations. Eur. Neuropsychopharmacol..

[62] Mahaffey K.W., Tuttle K.R., Arici M., Baeres F.M.M., Bakris G., Charytan D.M., Cherney D.Z.I., Chernin G., Correa-Rotter R., Gumprecht J. (2025). Cardiovascular outcomes with semaglutide by severity of chronic kidney disease in type 2 diabetes: The FLOW trial. Eur. Heart J..

[63] Li W., Liang X., Sun N., Zhang D. (2025). Influence of glucagon-like peptide-1 receptor agonists on renal parameters: A meta-analysis of randomized controlled trials. BMC Endocr. Disord..

[64] Sourris K.C., Ding Y., Maxwell S.S., Al-sharea A., Kantharidis P., Mohan M., Rosado C.J., Penfold S.A., Haase C., Xu Y. (2024). Glucagon-like peptide-1 receptor signaling modifies the extent of diabetic kidney disease through dampening the receptor for advanced glycation end products–induced inflammation. Kidney Int..

[65] Chen J., Dong X., Lin Y., Lv C. (2025). The critical role of GLP-1 signaling pathways in the pathology of Parkinson’s disease and diabetes. Pathol.—Res. Pr..

[66] Siegel R.L., Giaquinto A.N., Jemal A. (2024). Cancer statistics, 2024. CA Cancer J. Clin..

[67] Murphy A., Shyanti R.K., Mishra M. (2025). Targeting obesity, metabolic syndrome, and prostate cancer: GLP-1 agonists as emerging therapeutic agents. Discov. Oncol..

[68] Santoro A., McGraw T.E., Kahn B.B. (2021). Insulin action in adipocytes, adipose remodeling, and systemic effects. Cell Metab..

[69] Cancel M., Pouillot W., Mahéo K., Fontaine A., Crottès D., Fromont G. (2022). Interplay between Prostate Cancer and Adipose Microenvironment: A Complex and Flexible Scenario. Int. J. Mol. Sci..

[70] Hu X., Hu C., Zhang C., Zhang M., Long S., Cao Z. (2019). Role of Adiponectin in prostate cancer. Int. Braz. J. Urol..

[71] Armstrong M.J., Hull D., Guo K., Barton D., Hazlehurst J.M., Gathercole L.L., Nasiri M., Yu J., Gough S.C., Newsome P.N. (2016). Glucagon-like peptide 1 decreases lipotoxicity in non-alcoholic steatohepatitis. J. Hepatol..

[72] Pallegar N.K., Christian S.L., Birbrair A. (2020). Adipocytes in the Tumour Microenvironment. Tumor Microenvironment.

[73] Karzai F.H., Madan R.A., Dahut W.L. (2016). Metabolic Syndrome in Prostate Cancer: Impact on Risk and Outcomes. Future Oncol..

[74] Nauck M.A., Jensen T.J., Rosenkilde C., Calanna S., Buse J.B., The LEADER Publication Committee on behalf of the LEADER Trial Investigators (2018). Neoplasms Reported With Liraglutide or Placebo in People With Type 2 Diabetes: Results From the LEADER Randomized Trial. Diabetes Care.

[75] Levy S., Attia A., Elshazli R.M., Abdelmaksoud A., Tatum D., Aiash H., Toraih E.A. (2024). Differential Effects of GLP-1 Receptor Agonists on Cancer Risk in Obesity: A Nationwide Analysis of 1.1 Million Patients. Cancers.

[76] Chen P.H., Hibler E.A. (2023). Abstract 735: The associations between the use of GLP-1 receptor agonists, cancer recurrence and all-cause mortality among cancer survivors. Cancer Res..

[77] Eftekhari S., Montazeri H., Tarighi P. (2020). Synergistic anti-tumor effects of Liraglutide, a glucagon-like peptide-1 receptor agonist, along with Docetaxel on LNCaP prostate cancer cell line. Eur. J. Pharmacol..

[78] Du H., Meng X., Yao Y., Xu J. (2022). The mechanism and efficacy of GLP-1 receptor agonists in the treatment of Alzheimer’s disease. Front. Endocrinol..

[79] Scheltens P., De Strooper B., Kivipelto M., Holstege H., Chételat G., Teunissen C.E., Cummings J., Van der Flier W.M. (2021). Alzheimer’s disease. Lancet.

[80] Xie Y., Choi T., Al-Aly Z. (2025). Author Correction: Mapping the effectiveness and risks of GLP-1 receptor agonists. Nat. Med..

[81] Colin I.M., Szczepanski L.W., Gérard A.C., Elosegi J.A. (2023). Emerging Evidence for the Use of Antidiabetic Drugs, Glucagon-like Peptide 1 Receptor Agonists, for the Treatment of Alzheimer’s Disease. Eur. Endocrinol..

[82] Siddeeque N., Hussein M.H., Abdelmaksoud A., Bishop J., Attia A.S., Elshazli R.M., Fawzy M.S., Toraih E.A. (2024). Neuroprotective effects of GLP-1 receptor agonists in neurodegenerative Disorders: A Large-Scale Propensity-Matched cohort study. Int. Immunopharmacol..

[83] Delvadia P., Dhote V., Mandloi A.S., Soni R., Shah J. (2025). Dual GLP-1 and GIP Agonist Tirzepatide Exerted Neuroprotective Action in a Parkinson’s Disease Rat Model. ACS Chem. Neurosci..

[84] Nogueira L.O.S., Mazetto R.A.S.V., Defante M.L.R., Antunes V.L.J., Gonçalves O.R., Corso A.M.S., Della Coletta M.V., Boone D.L., Filho W.S.M., Borges V. (2025). Efficacy and safety of glucagon-like peptide 1 agonists for Parkinson’s disease: A systematic review and meta-analysis. Arq. Neuropsiquiatr..

[85] Helal M.M., AbouShawareb H., Abbas O.H., Haddad R., Zain Y., Osman A.S.A., Hassan A.K. (2025). GLP-1 receptor agonists in Parkinson’s disease: An updated comprehensive systematic review with meta-analysis. Diabetol. Metab. Syndr..

[86] Gamborg M., Grand M.K., Arvedsen J., Meaidi A., Mørch L.S. (2025). GLP -1 Agonists as Potential Neuromodulators in Development of Parkinson’s Disease: A Nationwide Cohort Study. Eur. J. Neurol..

[87] Tang H., Lu Y., Okun M.S., Donahoo W.T., Ramirez-Zamora A., Wang F., Huang Y., Armstrong M., Svensson M., Virnig B.A. (2024). Glucagon-Like Peptide-1 Receptor Agonists and Risk of Parkinson’s Disease in Patients with Type 2 Diabetes: A Population-Based Cohort Study. Mov. Disord..

[88] Aviles-Olmos I., Espinoza-Vinces C., Portugal L.R., Luquin M.R. (2025). Targeting Metabolic Dysfunction in Parkinson’s Disease: The Role of GLP-1 Agonists in Body Weight Regulation and Neuroprotection. Curr. Diab Rep..

[89] Moțățăianu A., Mănescu I.B., Șerban G., Bărcuțean L., Ion V., Bălașa R., Andone S. (2024). Exploring the Role of Metabolic Hormones in Amyotrophic Lateral Sclerosis. Int. J. Mol. Sci..

[90] Arai T., Atsukawa M., Tsubota A., Oikawa T., Tada T., Matsuura K., Ishikawa T., Abe H., Kato K., Morishita A. (2024). Beneficial effect of oral semaglutide for type 2 diabetes mellitus in patients with metabolic dysfunction-associated steatotic liver disease: A prospective, multicentre, observational study. Diabetes Obes. Metab..

[91] Newsome P.N., Buchholtz K., Cusi K., Linder M., Okanoue T., Ratziu V., Sanyal A.J., Sejling A.S., Harrison S.A. (2021). A Placebo-Controlled Trial of Subcutaneous Semaglutide in Nonalcoholic Steatohepatitis. N. Engl. J. Med..

[92] Abdelmalek M.F., Harrison S.A., Sanyal A.J. (2024). The role of glucagon-like peptide-1 receptor agonists in metabolic dysfunction-associated steatohepatitis. Diabetes Obes. Metab..

[93] Alkhouri N., Charlton M., Gray M., Noureddin M. (2025). The pleiotropic effects of glucagon-like peptide-1 receptor agonists in patients with metabolic dysfunction-associated steatohepatitis: A review for gastroenterologists. Expert Opin. Investig. Drugs.

[94] Bernsmeier C., Meyer-Gerspach A.C., Blaser L.S., Jeker L., Steinert R.E., Heim M.H., Beglinger C. (2014). Glucose-Induced Glucagon-Like Peptide 1 Secretion Is Deficient in Patients with Non-Alcoholic Fatty Liver Disease. PLoS ONE.

[95] Wang L., Berger N.A., Kaelber D.C., Xu R. (2024). Association of GLP-1 Receptor Agonists and Hepatocellular Carcinoma Incidence and Hepatic Decompensation in Patients With Type 2 Diabetes. Gastroenterology.

[96] Liu C., Zhang Q., Zhou H., Jin L., Liu C., Yang M., Zhao X., Ding W., Xie W., Kong H. (2024). GLP-1R activation attenuates the progression of pulmonary fibrosis via disrupting NLRP3 inflammasome/PFKFB3-driven glycolysis interaction and histone lactylation. J. Transl. Med..

[97] Malhotra A., Grunstein R.R., Fietze I., Weaver T.E., Redline S., Azarbarzin A., Sands S.A., Schwab R.J., Dunn J.P., Chakladar S. (2024). Tirzepatide for the Treatment of Obstructive Sleep Apnea and Obesity. N. Engl. J. Med..

[98] Beccuti G., Bioletto F., Parasiliti-Caprino M., Benso A., Ghigo E., Cicolin A., Broglio F. (2024). Estimating Cardiovascular Benefits of Tirzepatide in Sleep Apnea and Obesity: Insight from the SURMOUNT-OSA Trials. Curr. Obes. Rep..

[99] Meurot C., Jacques C., Martin C., Sudre L., Breton J., Rattenbach R., Bismuth K., Berenbaum F. (2022). Targeting the GLP-1/GLP-1R axis to treat osteoarthritis: A new opportunity?. J. Orthop. Transl..

[100] Baser O., Rodchenko K., Vivier E., Baser I., Lu Y., Mohamed M. (2024). The impact of approved anti-obesity medications on osteoarthritis. Expert Opin. Pharmacother..

[101] Singh M.P., Yadav R., Singh A. (2025). Efficacy and Safety of GLP-1 Receptor Agonists for the Management of Knee Osteoarthritis: A Systematic Review and Meta-Analysis. SN Compr. Clin. Med..

[102] Salamun V., Jensterle M., Janez A., Vrtacnik Bokal E. (2018). Liraglutide increases IVF pregnancy rates in obese PCOS women with poor response to first-line reproductive treatments: A pilot randomized study. Eur. J. Endocrinol..

[103] Bednarz K., Kowalczyk K., Cwynar M., Czapla D., Czarkowski W., Kmita D., Nowak A., Madej P. (2022). The Role of Glp-1 Receptor Agonists in Insulin Resistance with Concomitant Obesity Treatment in Polycystic Ovary Syndrome. Int. J. Mol. Sci..

[104] Bader S., Bhatti R., Mussa B., Abusanana S. (2024). A systematic review of GLP-1 on anthropometrics, metabolic and endocrine parameters in patients with PCOS. Women’s Health.

[105] Austregésilo De Athayde De Hollanda Morais B., Martins Prizão V., De Moura De Souza M., Ximenes Mendes B., Rodrigues Defante M.L., Cosendey Martins O., Rodrigues A.M. (2024). The efficacy and safety of GLP-1 agonists in PCOS women living with obesity in promoting weight loss and hormonal regulation: A meta-analysis of randomized controlled trials. J. Diabetes Complicat..

[106] Monney M., Mavromati M., Leboulleux S., Gariani K. (2025). Endocrine and metabolic effects of GLP-1 receptor agonists on women with PCOS, a narrative review. Endocr. Connect..

[107] Siamashvili M., Davis S.N. (2021). Update on the effects of GLP-1 receptor agonists for the treatment of polycystic ovary syndrome. Expert Rev. Clin. Pharmacol..

[108] Abdalla M.A., Deshmukh H., Atkin S., Sathyapalan T. (2021). The potential role of incretin-based therapies for polycystic ovary syndrome: A narrative review of the current evidence. Ther. Adv. Endocrinol. Metab..

[109] Elkind-Hirsch K., Marrioneaux O., Bhushan M., Vernor D., Bhushan R. (2008). Comparison of Single and Combined Treatment with Exenatide and Metformin on Menstrual Cyclicity in Overweight Women with Polycystic Ovary Syndrome. J. Clin. Endocrinol. Metab..

[110] Frangie Machado M., Shunk T., Hansen G., Harvey C., Fulford B., Hauf S., Schuh O., Kaldas M., Arcaroli E., Ortiz J. (2024). Clinical Effects of Glucagon-Like Peptide-1 Agonist Use for Weight Loss in Women with Polycystic Ovary Syndrome: A Scoping Review. Cureus.

[111] Salvio G., Ciarloni A., Ambo N., Bordoni M., Perrone M., Rossi S., Balercia G. (2025). Effects of glucagon-like peptide 1 receptor agonists on testicular dysfunction: A systematic review and meta-analysis. Andrology.

[112] La Vignera S., Condorelli R.A., Calogero A.E., Cannarella R., Aversa A. (2023). Sexual and Reproductive Outcomes in Obese Fertile Men with Functional Hypogonadism after Treatment with Liraglutide: Preliminary Results. J. Clin. Med..

[113] Yuan P., Ma D., Gao X., Wang J., Li R., Liu Z., Wang T., Wang S., Liu J., Liu X. (2020). Liraglutide Ameliorates Erectile Dysfunction via Regulating Oxidative Stress, the RhoA/ROCK Pathway and Autophagy in Diabetes Mellitus. Front. Pharmacol..

[114] Zhang H., Meng J., Li X., Zhou S., Qu D., Wang N., Jia M., Ma X., Luo X. (2015). Pro-GLP-1, a Pro-drug of GLP-1, is neuroprotective in cerebral ischemia. Eur. J. Pharm. Sci..

[115] Tamilwanan S., Aziz Z., Rong L.Y., Bitar A.N., Zarzour R.H.A., Alshehade S.A. (2025). Efficacy of GLP-1 receptor agonists and dual GLP-1/GIP receptor agonists in managing MALFD: A meta-analysis of randomized controlled trials. BMC Gastroenterol..

[116] Chiang C.H., Jaroenlapnopparat A., Colak S.C., Yu C.C., Xanthavanij N., Wang T.H., See X.Y., Lo S.W., Ko A., Chang Y.C. (2025). Glucagon-Like Peptide-1 Receptor Agonists and Gastrointestinal Adverse Events: A Systematic Review and Meta-Analysis. Gastroenterology.

